# Incarceration is associated with used syringe lending among active injection drug users with detectable plasma HIV-1 RNA: a longitudinal analysis

**DOI:** 10.1186/1471-2334-13-565

**Published:** 2013-12-01

**Authors:** M-J Milloy, Thomas Kerr, Kate Salters, Hasina Samji, Silvia Guillemi, Julio Montaner, Evan Wood

**Affiliations:** 1 British Columbia Centre for Excellence in HIV/AIDS, St. Paul’s Hospital, Vancouver, British Columbia, Canada; 2 Faculty of Medicine, University of British Columbia, Vancouver, British Columbia, Canada

## Abstract

**Background:**

Informed by recent studies demonstrating the central role of plasma HIV-1 RNA viral load (VL) on HIV transmission, interventions to employ HIV antiretroviral treatment as prevention (TasP) are underway. To optimize these efforts, evidence is needed to identify factors associated with both non-suppressed VL and HIV risk behaviours. Thus, we sought to assess the possible role played by exposure to correctional facilities on VL non-suppression and used syringe lending among HIV-seropositive people who use injection drugs (PWID).

**Methods:**

We used data from the ACCESS study, a community-recruited prospective cohort. We used longitudinal multivariate mixed-effects analyses to estimate the relationship between incarceration and plasma HIV-1 RNA > 500 copies/mL among antiretroviral therapy (ART)-exposed active PWID and, during periods of non-suppression, the relationship between incarceration and used syringe lending.

**Results:**

Between May 1996 and March 2012, 657 ART-exposed PWID were recruited. Incarceration was independently associated with higher odds of VL non-suppression (Adjusted Odds Ratio [AOR] = 1.54, 95% Confidence Interval [95% CI]: 1.10, 2.16). In a separate multivariate model restricted to periods of VL non-suppression, incarceration was independently associated with lending used syringes (AOR = 1.81, 95% CI: 1.03, 3.18).

**Conclusions:**

The current findings demonstrate that incarceration is associated with used syringe lending among active PWID with detectable plasma HIV-1 RNA. Our results provide a possible pathway for the commonly observed association between incarceration and increased risk of HIV transmission. Our results suggest that alternatives to incarceration of non-violent PWID and evidence-based combination HIV prevention interventions for PWID within correctional facilities are urgently needed.

## Background

Recent studies have demonstrated that plasma HIV-1 RNA viral loads (VL), at the individual, community and population levels, likely play a central role in determining HIV transmission dynamics. First demonstrated in studies of the role of maternal VL on the risk of perinatal vertical transmission [1], the link between VL and the incidence of HIV seroconversion has since been observed in observational analyses [2-4] and simulation models [5-7], and confirmed in the landmark HPTN-052 randomized clinical trial [8].

Informed by this insight, HIV treatment as prevention (TasP) has been adopted as a backbone of combination HIV prevention efforts [9,10] and public health-based efforts to seek, test, treat and retain (STTR) individuals in evidence-based medical care are underway. Evidence is now required to optimize TasP programmes in order to maximize their impact on HIV incidence. This is especially true among people who use injection drugs, who continue to experience high levels of HIV/AIDS-associated morbidity and mortality and account for more than one-quarter of incident cases outside sub-Saharan Africa [11]. Since there are concerns that TasP efforts might be compromised by, for example, difficulties detecting acutely-infected individuals [1,12] or improving engagement in the HIV clinical care cascade [2-4,13], HIV prevention interventions will benefit from the identification of exposures that are associated with both uncontrolled VL and risk behaviours for onward HIV transmission (i.e., used syringe lending).

Among people who inject drugs (PWID), exposure to correctional facilities is common and has been consistently associated with heightened risk of sharing used syringes in custodial [14,15] and non-custodial [16] settings. Although many PWID cease injection while incarcerated, the routine lack of sterile syringes often means levels of syringe sharing are typically higher in correctional settings than in the community [8,17,18]. As a result, prison-based HIV transmission has been reported in the United States [9,10,19], Scotland [11,20], Australia [21], Russia [22] and other locations [23,24]. Among people living with HIV, exposure to correctional facilities has been associated with sub-optimal HIV treatment including lower access and adherence to antiretroviral therapy (ART) [25,26]. However, we are unaware of any study that has evaluated the possible effects of both incarceration on VL non-suppression and used syringe lending among PWID living with HIV infection. Thus, in these analyses, we sought to estimate the relationships between incarceration and VL non-suppression and incarceration and used syringe lending during periods of viremia.

## Methods

To evaluate these objectives, we used data from the AIDS Care Cohort to evaluate Exposure to Survival Services (ACCESS), an ongoing prospective observational cohort of illicit drug users living with HIV/AIDS in Vancouver, British Columbia, Canada. Described in detail elsewhere [3,27], recruitment for the cohort began in May, 1996, and focused on the city’s Downtown Eastside neighbourhood, a post-industrial area with an open drug market and high levels of illicit drug use, poverty and HIV infection. We used community-based recruitment methods including targeted solicitation of areas where PWID are known to frequent, such as harm reduction venues, low-barrier service providers and single-room occupancy (SRO) hotels. Individuals are eligible for inclusion in ACCESS if they are HIV seropositive, are aged ≥ 18 years, have used illicit drugs other than cannabinoids in the previous month. After individuals are screened for inclusion and offered enrollment in the study, they complete an informed consenting process in a private and secure room at the study office. During this process, the participant and a study representative review a standardized letter which explains the study, its hypotheses and protocols and the possible risks and benefits of participation. Only individuals who provide written informed consent are included in the study. At the baseline interview and every biannual interview thereafter, participants respond to an interviewer-administered questionnaire, are examined by a study nurse and provide blood for serologic analyses. At recruitment, participants provide their personal health number (PHN), a unique and persistent identifier issued for billing and tracking purposes to all residents of British Columbia by the government-run universal and no-cost medical system. All study activities were reviewed and approved by the Providence Health Care/University of British Columbia Research Ethics Board (REB) prior to the commencement of the study. The REB reviews the study annually, including any changes to, for example, the recruitment process and all study instruments.

Information gathered through the interview and examination process is augmented by data on HIV treatment and clinical outcomes through a confidential linkage with the British Columbia Centre for Excellence in HIV/AIDS (BCCfE), as described elsewhere [27]. The BCCfE provides ART and related care free of charge to all individuals living with HIV/AIDS in British Columbia. Through the linkage a complete retrospective and prospective clinical profile, including data on all ART dispensations, CD4+ cell counts and plasma HIV-1 RNA viral load (PVL) observations, is available for each participant.

In this study, we included all individuals who had received at least one day of antiretroviral medication prior to the baseline interview. ART-naïve individuals who initiated treatment were included from the next follow-up interview forward. Individuals without at least one CD4+ cell count and PVL observation within ± 180 days of the baseline interview were excluded from these analyses. As well, because our objective was to model the risk of forward HIV transmission via contaminated syringes, we excluded an individual’s follow-up period if they did not report any injection drug use in that period.

Using this analytic sample, we tested three hypotheses: First, during any six-month follow-up period, incarceration is associated with higher odds of exhibiting detectable VL, independent of possible confounding factors. If the first hypothesis is true, we will test the second hypothesis: the independent relationship between incarceration and VL non-suppression is mediated by adherence to ART. Finally, we will test the third hypothesis: incarceration is associated with higher odds of used syringe lending during periods of VL non-suppression, independent of possible confounding factors.

In the test of the first hypothesis, the primary outcome of interest was detectable VL in the previous six months, defined as >500 RNA copies/mL plasma. This value was used as it is the lowest level available throughout the study period and is the same as previous analyses of VL from our setting [28,29]. HIV clinical monitoring data was gathered using each participant’s PHN through the confidential linkage described above and included all VL and CD4+ measurements conducted through the study as well as any VL/CD4+ tests conducted outside of the study setting, for example, by a participant’s personal physician. Thus, we were able to include all VL observations conducted in both community and correctional settings. In the event that more than one VL observation was conducted in the prior six months, we included the median of all observations. The Roche Amplicor Monitor assay was used to determine VL from participant blood samples (Roche Molecular Systems, Pleasanton, California).

The primary explanatory variable was incarceration in a city jail, provincial prison or federal penitentiary overnight or longer in the six month period prior to the interview, consistent with previous studies [30,31]. To best estimate the effect of incarceration on the likelihood of VL non-suppression, we considered a range of secondary explanatory variables we hypothesized might confound this relationship. These variables were: Age (per year older); gender (female vs. male); Aboriginal ancestry (yes vs. no); heroin injection (≥ daily vs. < daily); cocaine injection (≥ daily vs. < daily); crack cocaine use (≥ daily vs. < daily); education (≥ high school diploma vs. < high school diploma); homelessness (yes vs. no); involvement in sex work (yes vs. no); binge drug use, defined as any period of above-average drug use (yes vs. no); and methadone maintenance therapy (yes vs. no). All of the behavioural measures were time-updated and referred to the six month period prior to the follow-up interview, except for homelessness and MMT, which referred to current status. In addition, we included four clinical variables: HIV MD experience (< 6 patients vs. ≥ 6 patients previously treated for HIV); CD4+ cell count (per 100 cells); adherence to ART; and the presence of a protease inhibitor (PI) in the first ART regimen (yes vs. no). As in previous research [32,33], our physician experience variable was based on how many individuals the participant’s physician had previously enrolled in the HIV/AIDS treatment registry at the time of initiating the participant on their first dose of ART. This time-invariant measure was dichotomized at six patients, as before [32,33]. We have previously demonstrated that this measure independently predicts VL suppression following ART initiation [33]. As with our measure of VL, we included all CD4+ observations conducted by study staff or personal physicians and calculated a mean of all observations in the previous six months or, if no observations were made, the most recent measure. The measure of adherence to antiretroviral therapy was the product of the number of days dispensed antiretroviral therapy in the previous 180 days over the number of days eligible in the previous 180 days, dichotomized at above or below 95%. We have used this validated measure of pharmacy refill extensively in previous research [30,34] and have shown it reliably predicts VL suppression [1,35-37] and survival [2-4,32].

To determine the final set of secondary explanatory variables to include in the multivariate model, we first estimated bivariate statistics for the relationships between VL non-suppression and each explanatory variable of interest over the entire study period using generalized linear mixed-effects analysis. This form of regression analysis was used to account for the correlation between variables gathered over time from the same individual and, through the use of random intercepts, the unobserved variation in the likelihood of non-suppression within each individual [38].

Next, using an *a priori*-defined procedure first described by Maldonado and Greenland [8,39], we constructed a multivariate model including the primary explanatory variable and all secondary explanatory variables with *p*-values < 0.4 in bivariate analyses, noting the value of the β coefficient associated with incarceration. We then fit a series of multivariate models each with one secondary explanatory variable removed, always noting the value of the β coefficient associated with incarceration. After fitting all possible models, we removed the secondary explanatory variable corresponding to the smallest relative change in the β coefficient for incarceration from further consideration and repeated the process. This continued until the smallest change from the full model exceeded 5%, thus preserving secondary explanatory variables with a greater relative impact in the relationship between incarceration and the likelihood of non-suppressed VL. This technique has been used in many studies to estimate the independent effect of a variable on an outcome of interest [9,10,30,40,41]. To account for HIV disease progression, and, in the early HAART era, the phenomenon of individuals discontinuing therapy at high CD4+ counts to relieve treatment side-effects, we included CD4+ cell count as an explanatory covariate in all multivariate models.

To test our second hypothesis, that adherence to ART mediated the relationship between incarceration and VL non-suppression, we calculated the Sobel test statistic, using β and Standard Errors from regression models of incarceration, adherence and VL non-suppression [11,42,43], as in a previous study of age, adherence and VL suppression [12,44].

To test our third hypothesis, that incarceration was associated with a greater risk of used syringe lending among individuals experiencing viremia, we constructed a second multivariate model using the procedure described above. The outcome of interest was reporting lending used syringes in the previous six month period, as described in previous analyses [13,45]. The primary explanatory variable was incarceration in the previous six months. Secondary explanatory variables considered were: Age (per year older); gender (female vs. male); Aboriginal ancestry (yes vs. no); homelessness (yes vs. no); education (≥ high school diploma vs. < high school diploma); formal employment (yes vs. no); sex work (yes vs. no); heroin injection (≥ daily vs. < daily); cocaine injection (≥ daily vs. < daily); crack cocaine use (≥ daily vs. < daily); binge drug use (yes vs. no); MMT (yes vs. no); CD4+ cell count (per 100 cells/mL). We also included a variable for the year of observation (> 2001 vs. ≤ 2001), as we have previously described how local policy changes beginning in 2001, including increasing the supply of syringes, removing the requirement individuals return one used syringe for every new syringe received and decentralizing the distribution of syringes, were associated with a reduction in HIV incidence [14,15,46].

As above, we calculated bivariate relationships between syringe lending and each secondary explanatory variable, retaining explanatory variables with *p*-values < 0.4 for inclusion in the full multivariate model. Using an *a priori* defined protocol, we fit a series of reduced multivariate models to identify secondary explanatory variables with greater relative influence on the effect of incarceration on used syringe lending. As a last step, we fit the final multivariate model to best estimate the independent effect of incarceration on used syringe lending among individuals with non-suppressed VL.

## Results

Between May, 1996 and March, 2012, 1,036 HIV-seropositive illicit drug users were recruited and completed at least one interview. Of these, 217 (20.9%) were ART-naïve at recruitment and throughout the study and were excluded. Of the remaining 819, 783 (95.6%) had complete clinical data within 180 days of recruitment and 657 (80.2%) had at least one period of active injection drug use and were included in these analyses.

The 657 participants completed 3929 baseline or follow-up interviews, equal to 1965 person-years of observation. The median number of interviews completed was 4 (inter-quartile range [IQR]: 2, 8) per participant, or 2.4 (0.6, 4.8) per participant. Among the 657 individuals, 455 (69.3%) had at least one period of VL non-suppression. In 3929 interviews during the study period, 198 interviews contained reports of used syringe lending from 111 participants. For each interview, the median number of days since a CD4+ cell count observation was 58 days (IQR: 26, 127) and 3798 (96.7%) interviews occurred within 365 days of a CD4+ cell count observation.

This analytic sample was comprised of 228 (34.7%) women and 245 (37.3%) individuals reporting Aboriginal ancestry. Select socio-demographic, behavioural, structural and clinical characteristics of the participants at the baseline interview stratified by VL suppression are presented in Table 1.

**Table 1:** Baseline characteristics of 657 ART-exposed active PWID in Vancouver, Canada stratified by plasma HIV-1 RNA viral load (VL) non-suppression, 1996 – 2012

**Characteristic**	**VL suppressed**	**VL non-suppressed**	**Odds ratio**	**95% CI**^**2**^	***p*-value**
	**153 (23.3)**	**504 (76.7)**			
	**n (%)**	**n (%)**			
Incarceration^3^					
No	227 (87.3)	312 (78.6)	1.00		
Yes	33 (12.7)	85 (21.4)	1.87	1.21, 2.90	0.005
Age					
Median (IQR)	45.3 (39.5 – 50.7)	37.9 (33.1 – 43.9)	0.98	0.98, 0.98	< 0.001
Gender					
Male	191 (73.5)	238 (60.0)	1.00		
Female	69 (26.5)	159 (40.0)	1.85	1.32, 2.60	< 0.001
Aboriginal ancestry					
No	172 (66.2)	240 (60.5)	1.00		
Yes	88 (33.8)	157 (39.5)	1.28	0.92, 1.77	0.161
Heroin injection					
< Daily	230 (88.5)	293 (73.8)	1.00		
≥ Daily	30 (11.5)	104 (26.2)	2.72	1.75, 4.23	< 0.001
Cocaine injection					
< Daily	227 (87.3)	289 (72.8)	1.00		
≥ Daily	33 (12.7)	108 (27.2)	2.57	1.68, 3.94	< 0.001
Crack cocaine use					
< Daily	164 (63.1)	291 (73.3)	1.00		
≥ Daily	96 (36.9)	106 (26.7)	0.62	0.44, 0.87	0.007
Education					
< High school diploma	136 (52.3)	252 (63.5)	1.00		
≥ High school diploma	124 (47.7)	145 (36.5)	0.63	0.46, 0.87	0.005
Homeless					
No	243 (93.5)	352 (88.7)	1.00		
Yes	17 (6.5)	45 (11.3)	1.83	1.02, 3.27	0.041
Sex work^3^					
No	235 (90.4)	328 (82.6)	1.00		
Yes	25 (9.6)	69 (17.4)	1.98	1.21, 3.22	0.006
HIV MD experience					
≥ 6 patients	223 (85.8)	320 (80.6)	1.00		
< 6 patients	37 (14.2)	77 (19.4)	1.45	0.95, 2.22	0.093
PI in first ART regimen					
No	160 (61.5)	238 (59.9)	1.00		
Yes	100 (38.5)	150 (40.1)	1.07	0.78, 1.47	0.744
CD4+ cells (per 100/mm^3^)					
Median (IQR)	3.6 (2.4 – 4.9)	2.6 (1.5 – 4.0)	0.95	0.94, 0.97	< 0.001
ART adherence					
≤ 95%	102 (39.2)	377 (95.0)	1.00		
> 95%	158 (60.8)	20 (5.0)	0.03	0.02, 0.06	< 0.001

^2^95% Confidence Interval (95% CI).

^3^Refers to 180 day period prior to baseline interview.

The first hypothesis evaluated was that incarceration was associated with VL non-suppression independent of relevant confounders. There were 8,284 VL observations made over the entire study period, or a median of 9 (IQR: 4, 17) per participant, of which 3,314 (40.0%) indicated 500 HIV-1 RNA copies or greater per mL plasma. Non-suppression of VL was observed in 1972 (50.2%) of 3929 interviews. The crude and adjusted longitudinal estimates of the odds of VL non-suppression are presented in Table 2. In addition, in a sub-analysis, we observed a significant relationship between VL non-suppression and reporting used syringe lending (OR = 1.99, 95% CI: 1.30, 3.05, p = 0.002).

**Table 2:** Crude and adjusted longitudinal estimates of the odds of plasma HIV-1 RNA viral load non-suppression among 657 ART-exposed active PWID, Vancouver, 1996 – 2012

**Characteristic**	**OR**^**1**^	**95% CI**^**2**^	***p*-value**	**AOR**^**3**^	**95% CI**	***p*-value**
Incarceration^4^						
Yes vs. no	2.00	1.47 – 2.72	< 0.001	1.54	1.10, 2.16	0.011
Age						
Per year	0.90	0.87 – 0.92	< 0.001	0.91	0.88, 0.94	< 0.001
Gender						
Female vs. male	1.69	1.07 – 2.67	0.024			
Aboriginal ancestry						
Yes vs. no	1.35	0.86 – 2.11	0.189			
Homeless^5^						
Yes vs. no	2.38	1.59 – 3.58	< 0.001	1.83	1.19, 2.82	0.006
Education						
≥HS vs < HS	0.89	0.66 – 1.22	0.476			
Employment^4^						
Yes vs. no	0.48	0.25 – 0.92	0.028			
Heroin injection^4^						
≥ Daily vs. < daily	2.76	2.07 – 3.69	< 0.001	2.09	1.53, 2.87	<0.001
Cocaine injection^4^						
≥ Daily vs. < daily	1.52	1.18 – 1.97	0.001			
Crack cocaine use^4^						
≥ Daily vs. < daily	1.37	1.06 – 1.76	0.015			
Binge drug use^4^						
Yes vs. no	1.09	0.88 – 1.36	0.426			
MMT^5^						
Yes vs. no	0.46	0.35 – 0.59	< 0.001	0.52	0.39, 0.70	< 0.001
Sex work^4^						
Yes vs. no	2.21	1.47 – 3.33	< 0.001			
HIV MD experience						
< 6 patients vs. ≥ 6	1.93	1.08 – 3.46	0.026			
PI in first ART regimen						
Yes vs. no	1.06	0.68 – 1.67	0.794			
CD4+ cell count^4^						
Per 100 cells/mL	0.48	0.44 – 0.53	< 0.001	0.51	0.47, 0.56	< 0.001
ART adherence^4^						
≥ 95% vs < 95%	0.03	0.02 – 0.04	< 0.001			

^1^Odds Ratio; ^2^95% Confidence Interval; ^3^Adjusted Odds Ratio; ^4^Refers to the six month period prior to follow-up; ^5^Refers to current status.

Over the entire study period, incarceration was common, with 594 (15.1%) of all interviews including a report of at least one incarceration event in the previous six months. Among all participants, 235 (35.8%) were incarcerated at least once; among individuals ever incarcerated, the median number of periods including incarceration was 2 (IQR = 1, 3). Individuals incarcerated during the previous six months had at least double the odds of VL non-suppression compared to non-incarcerated individuals (OR = 2.00, 95% CI: 1.47, 2.72, *p* < 0.001). In a multivariate model adjusted for age, CD4+ cell count, engagement in MMT, heroin injection and homelessness, incarceration was independently associated with higher odds of VL non-suppression (Adjusted Odds Ratio = 1.54, 95% CI: 1.10, 2.16, p = 0.011). In a sub-analysis, we repeated the protocol adding year of observation to the model-building protocol (results not shown). Our results were not significantly different.

The second hypothesis was that ART adherence mediated the relationship between incarceration and VL non-suppression. A statistical test of mediation was significant (Sobel test = 4.09, *p* < 0.001).

The third hypothesis was that incarceration was associated with used syringe lending during periods of VL non-suppression, independent of potential confounders. Of the 198 interviews with a report of used syringe lending, 132 (66.7%) occurred during periods of VL non-suppression. Crude and adjusted longitudinal estimates of factors associated with used syringe lending among 455 active PWID with non-suppressed VL are presented in Table 3. Compared to non-incarcerated individuals, individuals reporting incarceration had almost double the odds of used syringe lending (OR = 1.76, 95% CI: 1.01, 3.05, *p* = 0.046). In the multivariate model, incarceration was independently associated with lending used syringes (AOR = 1.81, 95% CI: 1.03, 3.18, *p* = 0.038).

**Table 3:** Crude and adjusted longitudinal estimates of the odds of used syringe lending among 455 ART-exposed active PWID with unsuppressed plasma HIV-1 RNA viral loads, Vancouver, Canada, 1996 – 2012

**Characteristic**	**OR**^**1**^	**95% CI**^**2**^	***p*-value**	**AOR**^**3**^	**95% CI**	***p*-value**
Incarceration^4^						
Yes vs. no	1.76	1.01 – 3.05	0.046	1.81	1.03, 3.18	0.038
Age^5^						
Per year	1.03	0.94 – 1.13	0.515			
Gender						
Female vs. male	1.09	0.23 – 5.08	0.916			
Aboriginal ancestry						
Yes vs. no	0.30	0.09 – 0.96	0.043			
Homeless^5^						
Yes vs. no	1.18	0.40 – 3.47	0.759			
Education^5^						
≥ HS vs. < HS	0.82	0.36 – 1.86	0.635			
Employment^4^						
Yes vs. no	3.55	0.53 – 23.99	0.193			
Heroin injection^4^						
≥ Daily vs. < daily	0.77	0.35 – 1.70	0.520			
Cocaine injection^4^						
≥ Daily vs. < daily	1.27	0.64 – 2.51	0.500			
Crack cocaine use^4^						
≥ Daily vs. < daily	0.86	0.40 – 1.84	0.691			
Binge drug use^4^						
Yes vs. no	1.28	0.72 – 2.29	0.397			
MMT^5^						
Yes vs. no	1.27	0.58 – 2.79	0.553			
Sex work^4^						
Yes vs. no	1.72	0.67 – 4.44	0.259			
Year of observation						
> 2001 vs ≤ 2001	0.11	0.03 – 0.36	< 0.001	0.15	0.08, 0.29	< 0.001
CD4+ cell count						
Per 100 cells/mL	1.17	0.95 – 1.44	0.142			

^1^Odds Ratio; ^2^95% Confidence Interval; ^3^Adjusted Odds Ratio; ^4^Refers to the six month period prior to follow-up; ^5^Refers to current status.

## Discussion

In these analyses of data from a long-running community-recruited cohort of active PWID, we observed a high level of VL non-suppression. Uncontrolled VL was observed in more than half of all baseline and follow-up interview periods; more than two-thirds of all participants experienced at least one period of uncontrolled VL. In a multivariate model adjusted for a range of clinical, behavioural and structural correlates of non-suppression, incarceration was independently associated with greater odds of VL non-suppression. In a second multivariate model among PWID at risk of onward transmission, incarceration was independently associated with a greater likelihood of used syringe lending.

As with all observational studies, we cannot exclude the possibility that the observed association between incarceration and VL non-suppression is influenced by residual confounding. However, a number of lines of evidence support a causal relationship between exposure to correctional facilities and elevated viral load. Although correctional systems select for populations marked by high levels of various risks for sub-optimal HIV treatment outcomes, such as more severe addiction, our findings persisted even after they were adjusted for a range of significant factors, including homelessness, high-intensity illicit drug use, age and immunologic status. Consistent with the hypothesis that incarceration may contribute to antiretroviral interruption, prior studies of virally-suppressed PWID on ART in Vancouver, BC [17,18,31], and Baltimore, Maryland [19,26] identified incarceration as a strong independent risk factor for virologic rebound. Second, our finding that incarceration-related VL non-suppression is driven by poorer ART adherence is consistent with a range of previous analyses. For example, we recently demonstrated that the number of incarceration episodes experienced longitudinally was strongly associated with non-adherence to prescribed treatment in a dose-dependent fashion [20,30], in line with previous work isolating transfer between correctional/non-correctional environments [21,25] and prison-related HIV stigma as barriers to optimal ART adherence [22,47].

Our findings should be considered in light of increasing efforts to implement HIV treatment as prevention. Globally, HIV incidence among PWID remains high despite a range of effective evidence-based HIV prevention interventions, including sterile syringe distribution, opioid substitution therapies and supervised injection facilities [25,26,48]. Although these interventions are, by and large, simple and cost-effective, coverage remains low in many jurisdictions as criminal justice-based interventions remain the dominant response to injection drug use [3,23,27]. There is a clear danger that this dynamic will be repeated and the effect of TasP-based efforts to curb elevated HIV incidence among PWID will be compromised by incarceration-related barriers to HIV treatment. To date, there has been little discussion of the effect of the criminalization of PWID on TasP implementation as concerns have focused on behavioural factors associated with adherence [27,49]. Our evidence of the role played by incarceration on VL and HIV risk behaviours is an example of why structural dynamics must be considered in all combination HIV prevention interventions, including TasP [30,31,50,51].

As the HIV/AIDS pandemic enters its fourth decade, correctional facilities remain important foci of HIV incidence and prevalence, especially in Canada, the United States, areas of the former Soviet Union and south and southeast Asia [24,32,52,53]. Although data is incomplete, the prevalence of HIV infection is typically several times higher in detained populations compared to the general population and surpasses 10% in several jurisdictions [32,52]. Although some studies have demonstrated impressive clinical gains for people who are incarcerated and living with HIV/AIDS [30,34,54-56], and studies are underway of STTR efforts within a number of correctional systems in the United States [57], several prison-related barriers to optimal HIV treatment are common in many jurisdictions, including sub-standard healthcare facilities, poor continuity of care between correctional and non-correctional health systems, lack of care for HIV-related co-morbidities such as addiction and an emphasis on public security over public health [24,58,59]. In Canada, although the most recently released infectious disease surveillance data from 2007 reported that 1.6 percent of male and 4.7 percent of female individuals detained in the federal penitentiary system in 2007 were living with HIV/AIDS [60], we are unaware of any formal efforts to prevent prison-based HIV transmission through STTR initiatives. Further, officials in many settings have consistently refused to implement any prison-based needle exchange programmes, despite clear evidence of ongoing illicit drug use and syringe sharing within prisons and repeated calls by people who use injection drugs, physicians, legal experts and advocates [61,62]. Our current findings suggest that investments in HIV prevention and treatment efforts may be undercut by the lack of HIV prevention and treatment in prisons, especially among people who use injection drugs.

This research has some limitations. First, no registries of HIV-seropositive PWID exist and thus our analytic sample cannot be seen as representative of all HIV-positive PWID in this setting or others. Second, as mentioned above, we cannot exclude the possible existence of unmeasured confounding, however we sought to minimize its influence by multivariate modeling and the use of data a large and long-running community-recruited prospective cohort of illicit drug users. Third, our measure of incarceration was based on self-report as administrative data was unavailable. Fourth, we were unable to determine whether VL measurements occurred prior to, during or following an incarceration event. Our primary outcome of interest was ascertained through data from the local comprehensive provider of HIV clinical monitoring in a setting with no financial barriers to healthcare and we are unaware of any reason individuals might differentially report incarceration history based on their viral load status.

## Conclusions

To conclude, we used data from an ongoing prospective cohort of HIV-seropositive PWID with comprehensive clinical monitoring data and found incarceration was an independently associated with both non-suppression of PVL and used syringe sharing. To our knowledge, this is the first study to exploit recent advances in knowledge of VL and risk behaviours to model the risks of onward HIV transmission among people living with HIV infection. Our findings re-emphasize the importance of considering the structural determinants of HIV transmission, especially in the new era of combination HIV prevention interventions involving traditional HIV prevention strategies with the use of antiretroviral therapy. They also highlight the urgent need to identify and remove incarceration-related barriers to effective HIV treatment to address persistently high levels of HIV incidence and HIV/AIDS-related morbidity and mortality among people who use injection drugs. Finally, our results suggest that alternatives to incarceration of non-violent PWID and evidence-based combination HIV prevention interventions for PWID within correctional facilities are urgently needed.

## Competing interests

The authors state that they have no competing financial or non-financial interests. Dr. Montaner has also received financial support from the International AIDS Society, United Nations AIDS Program, World Health Organization, National Institutes of Health Research-Office of AIDS Research, National Institute of Allergy & Infectious Diseases, The United States President’s Emergency Plan for AIDS Relief (PEPfAR), UNICEF, the University of British Columbia, Simon Fraser University, Providence Health Care and Vancouver Coastal Health Authority. He has received grants from Abbott, Boehringer-Ingelheim, Bristol-Myers Squibb, Gilead Sciences, Janssen, Merck and ViiV Healthcare.

## Authors’ contributions

M-JM, EW and TK conceived the analysis; M-JM conducted the statistical analysis and drafted and revised the manuscript; HS, KS, SG, JM, M-JM, EW and TK designed the study, coordinated the gathering of clinical, laboratory and interview data, reviewed and commented on the analysis and the manuscript. All authors read and approved the final version of the manuscript.

## Pre-publication history

The pre-publication history for this paper can be accessed here:

http://www.biomedcentral.com/1471-2334/13/565/prepub
